# Synthesis of some novel annulated pyrido[2,3-*d*]pyrimidines via stereoselective intramolecular hetero Diels–Alder reactions of 1-oxa-1,3-butadienes

**DOI:** 10.3762/bjoc.6.11

**Published:** 2010-02-04

**Authors:** Mohit L Deb, Pulak J Bhuyan

**Affiliations:** 1 Medicinal Chemistry Division, North East Institute of Science & Technology, Jorhat 785006, Assam, India, Fax: 0376 2370011

**Keywords:** β-halo aldehydes, hetero Diels–Alder reaction, 1-oxa-1,3-butadiene, pyrido[2,3-*d*]pyrimidines, uracil

## Abstract

Some novel annulated pyrido[2,3-*d*]pyrimidines **6** and **7** were synthesized stereoselectively by intramolecular hetero Diels–Alder reactions involving 1-oxa-1,3-butadienes.

## Introduction

The importance of uracil and its annulated derivatives is well recognized by synthetic as well as biological chemists [1–8]. Pyrido[2,3-*d*]pyrimidines represent a broad class of annelated uracils which have received considerable attention over the past years due to their wide range of biological activities such as antibacterial [9–10], antitumor [11–12], cardiotonic [13–14], hepatoprotective [13], antihypertensive [13], bronchiodilator [15] and vasodilator [16] properties. Additionally, some compounds of this type exhibit antialergic [17], antimalarial [18], analgesic [19–20] and antifungal [21] activity. Consequently, much effort has been directed towards the synthetic manipulation of uracil for the preparation of these complex molecules. However, there still remains many challenges in the synthesis of these naturally occurring complex molecules [22–31].

Hetero Diels–Alder reactions [32–35] are becoming a mainstay of heterocyclic and natural product synthesis. This powerful reaction method does not only allow the efficient synthesis of complex compounds starting from simple substrates but also permits the preparation of highly diversified molecules. One such reaction type, the oxabutadiene Diels–Alder reaction is a very useful method for the synthesis of dihydropyrans [36–38].

Heterocyclic β-halo aldehydes are very interesting compounds which can be transformed in a number of ways to fused heterocycles [39–40] by using the reactivity of halide for nucleophilic substitution in combination with a multitude of transformation possibilities from the aldehyde function.

## Results and Discussion

**Scheme 1:**
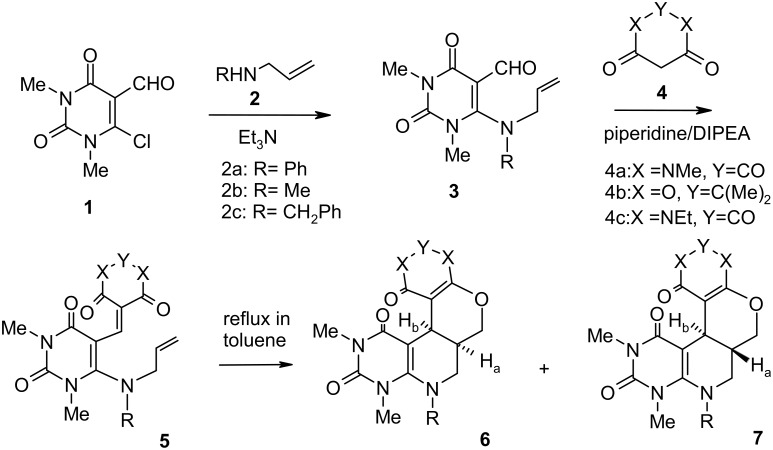
Stereoselective synthesis of some new complex annulated pyrido[2,3-*d*]pyrimidines by intramolecular hetero Diels–Alder reactions involving 1-oxa-1,3-butadienes.

As part of our continued interest in uracils [41–45] and the development of highly expedient methods for the synthesis of diverse heterocyclic compounds of biological importance [46–50], we now report the stereoselective synthesis of some new complex annulated pyrido[2,3-*d*]pyrimidines by intramolecular hetero Diels–Alder reactions involving 1-oxa-1,3-butadienes (Scheme 1).

The key intermediate, the 2-chloro-3-formyl uracil derivative **1** (β-halo aldehyde), was prepared by the reaction of *N,N*-dimethyl barbituric acid with Vielsmeier reagent (DMF + POCl_3_) using excess phosphorous oxychloride as solvent following our published method [51]. The nucleophilic substitution of the chloro group of **1** by allyl amines **2** afforded the 6-*N*-allyl-1,3-dimethyl-5-formyl uracils **3**. Compounds **3** were then reacted with cyclic β-diamides/β-diketones **4** in presence of a base catalyst (usually piperidine) to produce the 1-oxa-1,3-butadienes **5** which underwent intramolecular Diels–Alder reaction under reflux conditions in toluene (12 to 15 h) to give the cycloadducts. In most of cases the formation of two compounds was observed. The compounds were separated by column chromatography and their structures were determined from their spectroscopic data as the *cis*-(**6**) and *trans*-(**7**) isomers of the cycloadduct. The generality of the reaction was established by synthesizing a series of tetracyclic annulated uracil derivatives **6a**–**h** and **7a**–**e**. Our results are recorded in Table 1.

**Table 1:** Synthesis of some novel annulated pyrido[2,3-*d*]pyrimidines **6**/**7**.^a^

Entry	Allyl amines **(2)**	Compound **(4)**	Product yield^b^	Reaction time (h)	*cis* **:** *trans* **6 : 7**

1	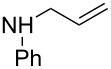	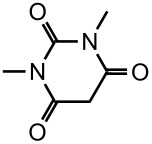	**6a** + **7a** (64%)	15	78 : 22
2	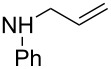	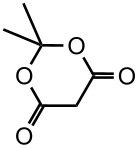	**6b** + **7b** (55%)	12	84 : 16
3	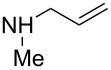	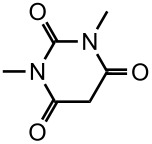	**6c** + **7c** (62%)	16	78 : 22
4	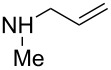	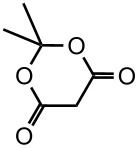	**6d** + **7d** (50%)	12	88 : 12
5	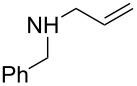	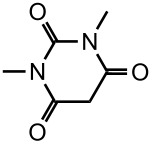	**6e** + **7e** (60%)	16	76 : 24
6	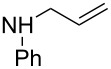	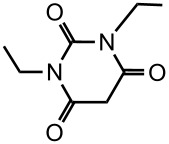	**6f** (62%)	15	100 : 0
7	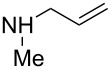	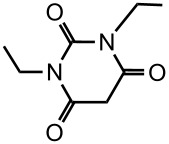	**6g** (60%)	16	100 : 0
8	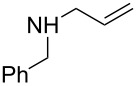	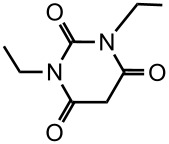	**6h** (57%)	16	100 : 0

^a^All the reactions were carried out under reflux conditions. ^b^Isolated yields.

In all of the reactions the *cis*-annelated products were formed either predominantly or exclusively. As the oxabutadiene is part of a cyclic compound and two carbon atoms to which the dienophile is attached are part of a ring system, the *endo*-transition state is energetically more favorable than the *exo*-transition state, and thus the *cis*-cycloadducts were formed predominantly (entries 1–5). In case of entries 6–8, the bulky ethyl group further favors the *endo*-transition and in these cases *cis*-annelated products were formed exclusively. In some of our previous efforts, we also obtained either *cis*-isomers or the mixtures of both the *cis*- and *trans*-isomers [52–53].

When *N*-methylallylamine (**2b**) or *N*-benzylallylamine (**2c**) was used for the reaction as shown in Scheme 1, the Knoevenagel condensation did not occur even after refluxing in the presence of piperidine. The reason might be the delocalization of the lone pair on the allyl nitrogen through the formyl group which makes it less electrophilic towards the active methylene compounds. However, the use of a stronger base, e.g. *N,N*-diisopropylethylamine (DIPEA) under refluxing conditions in ethanol for 3 h gave quite satisfactory results in all cases. In the case of *N*-phenylallylamine substituted uracils the condensation reactions proceeded normally in the presence of either of the bases.

## Conclusion

In summary, we report the stereoselective preparation of some new complex tetracyclic annulated uracil derivatives by intramolecular hetero Diels–Alder reactions involving 1-oxa-1,3-butadiene. This reaction, which can also be investigated for the synthesis of many other heyterocyclic compounds of biological importance, is a valuable addition to the chemistry of uracils.

## Experimental

All reagents and solvents were of reagent grade and were used without drying. The IR spectra were recorded on a Perkin Elmer system-2000 FTIR spectrometer. ^1^H NMR and ^13^C NMR spectra were recorded on Bruker Avance-DPX 300 MHz and 75 MHz FT NMR in CDCl_3_ using TMS as the internal standard. LR-MS were recorded on a Bruker Daltonics ESQUIRE 3000 LC ESI ion trap mass spectrometer and HRMS were obtained with a MALDI-TOF instrument. Elemental analyses were performed on a Perkin Elmer-2400 spectrometer. Analytical TLC and column chromatography were performed using E. Merck aluminum-backed silica gel plates coated with silica gel G and E. Merck silica gel (100–200 Mesh); melting points (uncorrected) were determined on a Büchi B-540 apparatus.

**Preparation of 1,3-dimethyl-6-chloro-5-formyluracil (1):** DMF (12 ml) in a 100 ml round bottomed flask was very slowly treated with phosphorous oxychloride (46 ml) with cooling after the addition of every 1 ml portion of POCl_3_. 1,3-Dimethylbarbituric acid (4 g) was added and the mixture heated under reflux for 1 h. Excess POCl_3_ was removed under reduced pressure. The viscous mixture was poured into ice-cold water and then extracted with dichloromethane (two to three times). After drying with sodium sulfate, the dichloromethane was removed under reduced pressure. The brown compound obtained contained some impurities and was used without further purification.

**Reaction of 1,3-dimethyl-6-chloro-5-formyluracil (1) with *N*-allylanilines/*N*-allylamines (2) and preparation of 6-amino-5-formyluracils 3:** Compound **1** (2 mmol, 404 mg) dissolved in dichloromethane, was treated with an equivalent amount of *N*-allylaniline (**2a**) (2 mmol, 266 mg) and triethylamine (2 mmol), and the mixture stirred at room temperature for 2 h. The solvent was evaporated and the residue was purified by column chromatography (silica gel, 100–200 Mesh) using dichloromethane to give **3a** as a yellow solid 568 mg (95%). Similarly, **3b**–**c** were prepared from the reaction of **1** with **2b**–**c**.

**Knoevenagel condensation of 3 with cyclic β-diamide/β-diketones 4 and synthesis of 5:** Equimolar amounts of **3a** (2 mmol, 598 mg) and **4a** (2 mmol, 312 mg) were mixed thoroughly in a round-bottomed flask containing water (8 ml). Two drops of piperidine (in case of Meldrum’s acid (**4b**), piperidine acetate was used) were added and the mixture stirred for 4 h. The solid was removed by filtration and recrystallized from ethanol to afford a white solid (769 mg, 88%). The compound was assigned structure **5a** from a consideration of its spectroscopic data. In case of *N*-methylallylamine **2b** and *N*-benzylallylamine **2c**, *N,N*-diisopropylethylamine (DIPEA) was used as catalyst for the Knoevenagel condensation and reactions were performed under reflux in ethanol for 3 h. The method gave satisfactory yields of up to 90%. Compounds **5b**–**h** were synthesized similarly.

**Intramolecular Diels–Alder reaction of compound 5:** Compound **5a** (1 mmol, 437 mg) was dissolved in toluene (6 ml) and heated under reflux for 15 h. After completion of the reaction (as monitored by TLC), the solvent was removed under reduced pressure. Two products (indicated by TLC) were separated by column chromatography using 65% ethyl acetate in petroleum ether. The structures were assigned from a combination of their spectral data and elemental analysis. From the value of coupling constant, it was established that the products **6a/7a** are *cis*-/*trans*-isomers, respectively. Total yield = 64% (280 mg). The other hetero Diels–Alder products **6b**–**h** and **7b**–**e** were synthesized similarly.

***cis*-isomer 6a:** mp 312–314 °C. IR (KBr); 3033, 2954, 1698, 1687, 1165, 744 cm^−1^. ^1^H NMR (300 MHz, CDCl_3_); δ, 2.96 (s, 3H), 2.98 (s, 3H), 3.21 (s, 3H), 3.47 (s, 3H), 3.59–3.68 (m, 3H), 4.23 (d, *J* = 5.28 Hz, 2H), 4.39 (d, *J* = 6.39 Hz, 1H), 7.0 (t, 2H, *J* = 7.41 Hz), 7.17–7.27 (m, 3H). ^13^C NMR (75 MHz, CDCl_3_); δ, 163.21, 156.35, 153.37, 149.54, 136.91, 129.95, 123.86, 118.69, 104.32, 89.61, 51.52, 37.05, 32.05, 29.0, 28.78, 28.31, 26.18. *m/z* 438.2 (M + H)^+^. Anal. Calcd for C_22_H_23_N_5_O_5_; C, 60.41; H, 5.26; N, 16.01; found C, 60.68; H, 5.33; N, 15.87.

***trans*-isomer 7a:** mp 317–318 °C. IR (KBr); 3033, 2954, 1698, 1687, 1165, 744 cm^−1^. ^1^H NMR (300 MHz, CDCl_3_); δ, 2.96 (s, 3H), 2.98 (s, 3H), 3.21 (s, 3H), 3.45 (s, 3H), 3.53–3.67 (m, 3H), 4.24 (d, *J* = 5.8 Hz, 2H), 4.36 (d, *J* = 11.7 Hz, 1H), 7.08 (t, 2H, *J* = 7.36 Hz), 7.17–7.27 (m, 3H). ^13^C NMR (75 MHz, CDCl_3_); δ, 163.26, 156.35, 153.30, 149.47, 136.91, 129.63, 123.86, 118.65, 105.47, 89.61, 51.31, 37.43, 32.05, 29.11, 28.78, 28.31, 26.18. *m/z* 438.2 (M + H)^+^. Anal. Calcd for C_22_H_23_N_5_O_5_; C, 60.41; H, 5.26; N, 16.01; found C, 60.65; H, 5.37; N, 15.82.

## Supporting Information

File 1 Spectroscopic and elemental analyses data of the compounds **6b–h** and **7b–e**.
